# Measurement of the retinal venous pressure with a new instrument in healthy subjects

**DOI:** 10.1007/s00417-021-05374-y

**Published:** 2021-09-09

**Authors:** Richard Stodtmeister, Emilie Wetzk, Robert Herber, Karin R. Pillunat, Lutz E. Pillunat

**Affiliations:** Univ. Klinikum Carl Gustav Carus, TU Dresden, Fetscherstrasse 74, 01307 Dresden, Germany

**Keywords:** Venous pressure, Retina, Dynamometry, Glaucoma, Valsalva manoeuvre

## Abstract

**Background:**

The retinal venous pressure (RVP) is a determining factor for the blood supply of the retina as well as the optic nerve head and until recently has been measured by contact lens dynamometry (CLD). A new method has been developed, potentially offering better acceptance. The applicability and the results of both methods were compared.

**Methods:**

The type of this study is cross sectional. The subjects were 36 healthy volunteers, age 26 ± 5 years (mean ± s). Tonometry: rebound tonometer (RT) (iCare). The measurements were performed during an increase in airway pressure of 20 mmHg (Valsalva manoeuvre). Principle of RVP measurement: the central retinal vein (CRV) is observed during an increase of intraocular pressure (IOP) and at the start of pulsation, which corresponds with the RVP. Two different instruments for the IOP enhancement where used: contact lens dynamometry and the new instrument, IOPstim. Principle: a deflated balloon of 8 mm diameter—placed on the sclera laterally of the cornea—is filled with air. As soon as a venous pulsation occurs, filling is stopped and the IOP is measured, equalling the RVP. Examination procedure: randomization of the sequence: CLD or IOPstim, IOP, mydriasis, IOP three single measurements (SM) of the IOP with RT or of the pressure increase with CLD at an airway pressure of 20 mmHg, 5 min break, IOP, and three SM using the second method at equal pressure (20 mmHg).

**Results:**

Spontaneous pulsation of the CRV was present in all 36 subjects. Pressures are given in mmHg. IOP in mydriasis 15.6 ± 3.3 (m ± s). Median RVP (MRVP)) of the three SM: CLD/IOPstim, 37.7 ± 5.2/24.7 ± 4.8 (*t* test: *p* < 0.001). Range of SM: 3.2 ± 1.8/2.9 ± 1.3 (*t* test: *p* = 0.36). Intraclass correlation coefficient (ICC) of SM: 0.88/0.83. ANOVA in SM: *p* = 0.48/0.08. MRVP CLD minus MRVP IOPstim: 13.0 ± 5.6. Ratio MRVP CLD/MRVP IOPstim: 1.56 ± 3.1. Cooperation and agreeability were slightly better with the IOPstim.

**Conclusion:**

This first study with the IOPstim in humans was deliberately performed in healthy volunteers using Valsalva conditions. As demonstrated by ICC and ANOVA, reproducible SM can be obtained by both methods and the range of the SM does not differ greatly. The higher MRVP in CLD could be explained by the different directions of the force vectors.

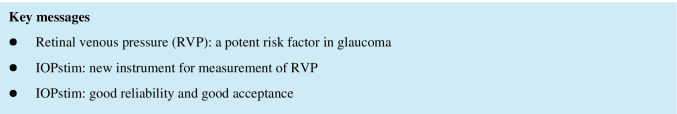

## Introduction

Bailliart first described the use of dynamometry to measure the blood pressure of the eye [1]. The term technically means the measurement of force. A force needs to be applied to the eye to induce a rise in intraocular pressure (IOP), which elicits pulsation of the retinal vessels on or near the optic disc. Different instruments have been invented: the first one was developed by Bailliart [1]; the impression dynamometer was developed by Müller [2]; and the concave lens dynamometer [3], the angiotonometer [2, 4], the suction cup dynamometer [5] and the contact lens dynamometer (CLD) have been developed [6]. With the exception of the CLD these instruments were calibrated in arbitrary units that had to be converted into intraocular pressure units. This conversion showed considerable variability [7]. To overcome this disadvantage, a new instrument was developed in which the artificially enhanced IOP is measured by commercially available tonometers that have been officially verified. The advantages of this instrument over many of the abovementioned instruments are that it can be handled by a single examiner and that it does not need official verification because the measurement itself is used for tonometry. In the present study, the instrument is described for the first time, and the retinal venous pressure measurement results obtained with this instrument are compared with those obtained by the CLD in healthy subjects. The applicability and reliability of the instrument in humans were also assessed.

## Methods

### Instruments

**Fig. 1:**
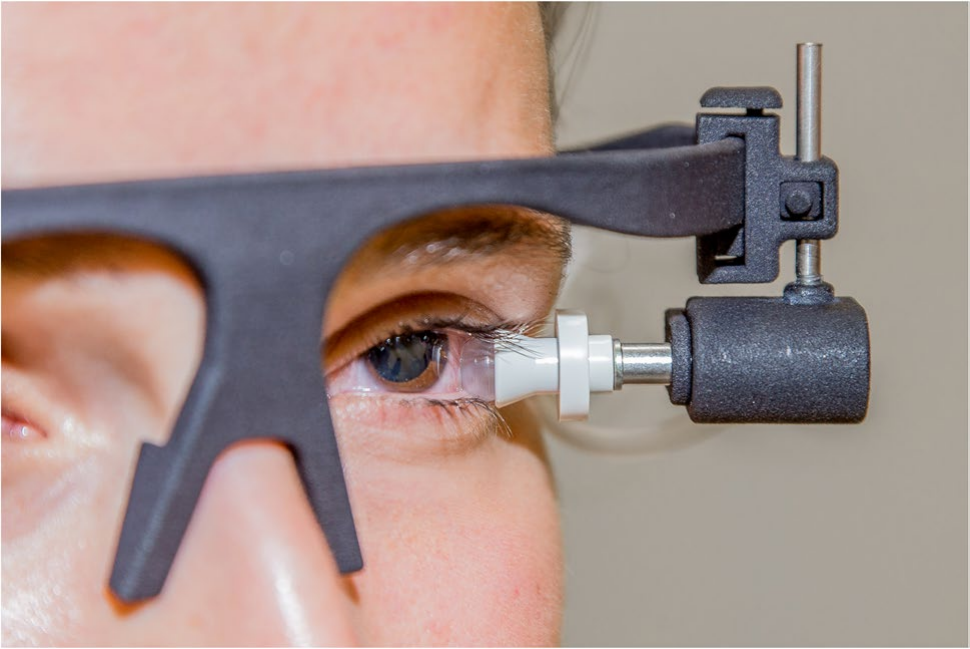
The IOPstim positioned at the eye. Copyright: TU Dresden.

**Fig. 2:**
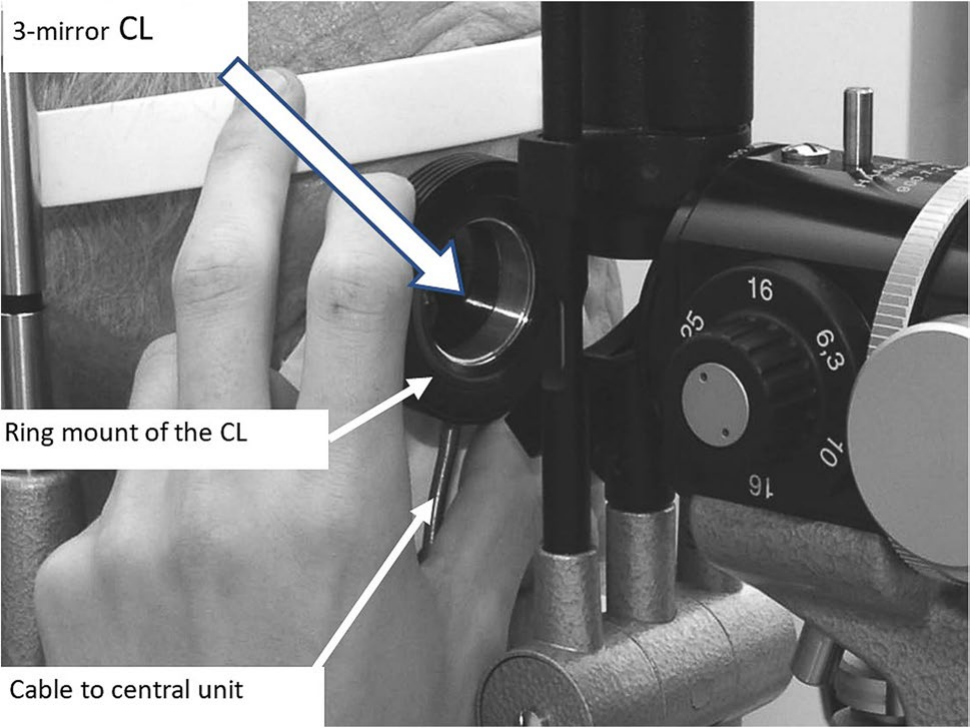
Measurement of the retinal venous pressure by contact lens (CL) dynamometry. The Goldmann 3-mirror CL is held in place by the ring mount which is connected with the CL by strain gauges whose signal is sent to the central unit by a flexible cable

The new instrument (Fig. 1) enhances the IOP by the inflation of a balloon of 8 mm diameter mounted on a cup. The balloon is positioned laterally to the cornea on the globe. Surface anaesthesia is recommended (Fig. 2). The expendable balloon is mounted on a spectacle-like frame and connected by a flexible silicone tube to a motor pump that is operated by a foot switch. The measurement process is as follows: the frame is mounted on the head of the subject, and the central unit with the motor pump is activated. At this stage, the balloon has the shape of a hemisphere due to its material stiffness. As soon as it touches the globe, its pressure is reduced to − 12 mmHg. Due to this pressure change, the balloon is pulled into the cup, which is positioned on the globe with a force that is as small as possible. Then, the central retinal vein (CRV) and its branches on the optic disc or close to it are inspected. In case one of these vessels pulsates, the IOP is measured by a commercially available tonometer, and the measurement value is noted as the retinal venous pressure (RVP). If there is still no pulsation, the motor pump is started to increase the pressure in the balloon. With this procedure, the balloon enlarges. This procedure in turn exerts force on the globe, resulting in an increase in the IOP. As soon as the CRV pulsates, the motor pump is stopped, the IOP is measured immediately, and the balloon is deflated. The measured IOP is the RVP.

The instrument is called IOPstim because it may stimulate the retinal vessels to pulsate. It is manufactured by Imedos Health GmbH in Jena, Germany.

### Contact lens dynamometry

The results obtained with this new method were compared with those obtained by a contact lens dynamometer (CLD). The CLD has, until now, been the method of choice to measure the RVP [6]. The instrument consists of a commercially available Goldmann 3-mirror contact lens that is connected by strain gauges to a metal ring. The signals of these sensors are processed in a central unit, and the result is displayed as an increase in the intraocular pressure. In the measurement of the retinal venous pressure, the instrument is attached to the eye, and the optic nerve head with its vessels is examined while the force of attachment is gradually increased. As soon as the CRV pulsates, the measurement is stopped, and the increase in intraocular pressure is read from the liquid crystal display on the central control unit. The RVP is calculated as the sum of the initial intraocular pressure and this reading. A more detailed description of the measurement procedure is given in an earlier publication [8].

### Subjects and procedure

This cross-sectional study was performed in accordance with the Declaration of Helsinki and was approved by the institutional ethics committee of the Technical University of Dresden (EK 322062019). The measurements were performed in 36 healthy Caucasian volunteers (Table 1) who provided informed consent. The inclusion criteria were as follows: healthy subjects aged 18–49 years with a pupil diameter in mydriasis of ≥ 6 mm. The exclusion criteria were as follows: extraocular and intraocular inflammation, retinal detachment, corneal scars, blurred optical media, monophthalmia, spherical refraction equivalent of < − 5 dioptres, arterial hypertension, nephropathia, diabetes mellitus, cold extremities, cardiopathies, earlier eye surgery, glaucoma and insufficient compliance. Every subject included in this study could be measured by both methods and no one had to be excluded because of insufficient compliance. The left eye was examined. The order in which the instruments were used (IOPstim or CLD) was determined by an urn model without replacement in groups of ten. The protocol was as follows: initial rebound tonometry (RT; iCare, Tiolat Oy, Vantaa, Finland), mydriasis, RT, semiautomatic systemic blood pressure measurement (Omron 5 Professional, Omron, Kyoto, Japan) and three measurements of RVP by the IOPstim or CLD in quick succession during the Valsalva manoeuvre (VM) at an airway pressure of 20 mmHg. During the VM, the subject blew into a flexible tube connected to an aneroid manometer, which is used for manual BP measurement (Fazzini, Vimodrone, Italy), positioned in front of the right eye, RT. The procedure was repeated with the second instrument.

**Table 1:** Description data of the subjects. *BPsys*, systolic blood pressure; *BPdia*, diastolic blood pressure; Init. *IOP*, intraocular pressure before mydriasis. *HF*, heart rate frequency, beats per minute; *BCVA*, best corrected visual acuity. Abbreviations: *Q1* first quartile, *Q3* third quartile, *IQR* interquartile range

*N* = 36						
m/f: 13/23						
	Min	Q1	Med	Q3	Max	IQR
Age, years	19.9	22.7	24.6	29.4	40.4	6.7
BPsys, mmHg	92	108	118	127	164	19
BPdia, mmHg	67	74	80	86	105	12
HF, bpm	55	66	72	79	98	13
BMI, kg/m^2^	18.7	20.7	21.8	23.8	29.0	3.0
Init. IOP, mmHg	11.8	15.2	16.4	18.1	24.4	2.9
BCVA, dec	0.8	1.0	1.0	1.0	1.3	0.0

The cooperation of the subjects was rated using 4 classes: (1) the lids were fully slack, indicating excellent cooperation; (2) there was low lid tension, indicating good cooperation; (3) it was very difficult to attach the instrument to the globe, indicating fair cooperation; (4) the examination was terminated early, indicating insufficient cooperation. Agreeability was assessed by a 5-stage classification system: (1) contact was sensed, and there was no irritation, indicating an excellent outcome; (2) contact was sensed, and it was well tolerated, indicating a good outcome; (3) contact was poor, and it was tolerated for some minutes only, indicating a fair outcome; (4) contact was painful, and it was nearly intolerable; (5) the examination disrupted because of pain. Parametric and non-parametric descriptive tests were performed, depending on the distribution of the variables. In the examination session, the patient was asked about the subjective comfort of the two methods, and the examiner reported which of the two methods was easier to handle.

The statistical analysis was performed using spreadsheet software (Excel 2016 software, Microsoft Corp.) to collect the data as well as SPSS (version 25, IBM Corp.) and Statistica (version 12.1SP1, Statsoft, Europe) to perform the statistical tests. The normality of the data was assessed by p-p diagrams. Different test methods were used according to whether the data were normally distributed. A *p* value lower than 0.05 was considered statistically significant. The repeatability of three consecutive measurements was assessed by the intraclass correlation coefficient.

## Results

**Fig. 3:**
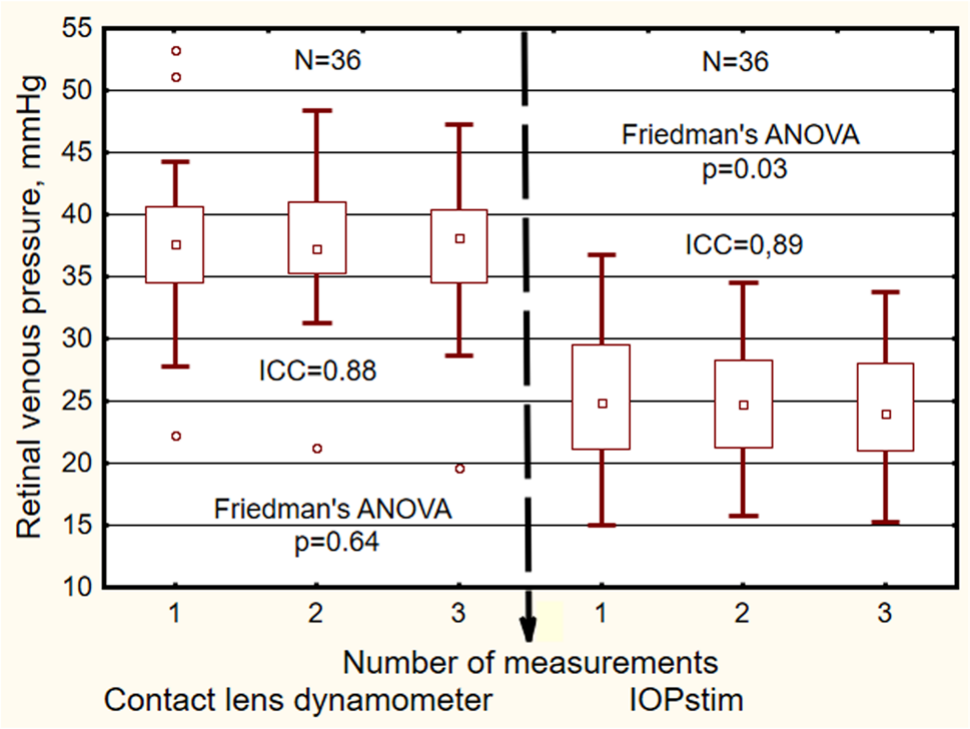
Box plots of RVP at the three measurement time points at an airway pressure of 20 mmHg. Abscissa: number of measurements. Left side: RVP measured by the CLD. Right side: RVP measured by the IOPstim. Ordinate: RVP. Abbreviations: RVP = retinal venous pressure. CLD = contact lens dynamometer. IOPstim = intraocular pressure stimulator

**Fig. 4:**
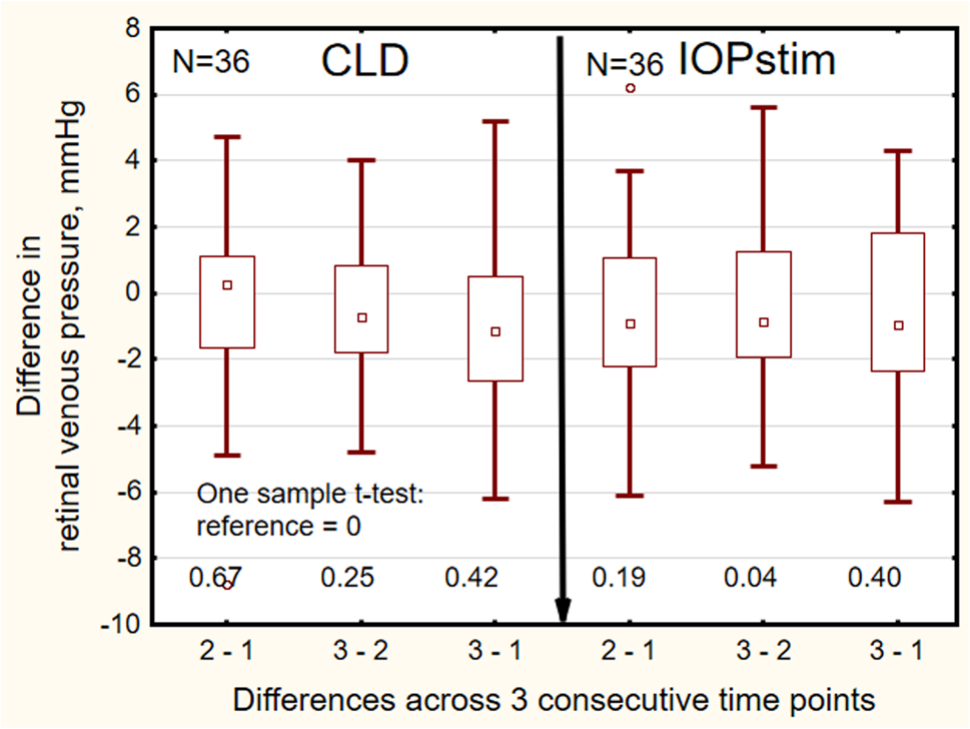
Differences in the retinal venous pressure (RVP) values taken in rapid succession by contact lens dynamometry (left) and by IOPstim (right). Abscissa: 2–1: Time point (TP) 2–TP 1; 3–2: TP 3–TP 2; 3–1: TP 3–TP 1. Ordinate: RVP difference in mmHg, negative values: decrease in the RVP. Airway pressure 20 mmHg. For abbreviations, see Fig. 2

The demographic characteristics of the study population are presented in Table 1. The RVP values (Table 2) measured by the CLD were evidently higher than the values obtained with the IOPstim (Fig. 3). The CLD/IOPstim ratio was 1.57 ± 0.31 (mean ± s). The three single *measurement values* obtained by CLD did not differ significantly (Friedman ANOVA: *p* = 0.64). However, as measured by the IOPstim, the median of the third value was 0.9 mmHg smaller (Friedman ANOVA: *p* = 0.03) (Fig. 3). For the three measurements determined by the CLD, the intraclass correlation coefficient was 0.88 (95% confidence interval: 0.801–0.931), and in the IOPstim measurements, it was 0.89 (95% confidence interval: 0.83–0.94). The median *differences* in the measurement values for one subject across the 3 single measurements (Fig. 4) did not differ significantly from zero with the CLD method (CLDM) (one sample *t* test, reference = 0: *p* = 0.25). There was a slight difference between the second and third measurements with the IOPstim method (IOPstimM) (*p* = 0.04). The median range of the three single measurements was 2.7 mmHg with the CLDM and 2.9 mmHg with the IOPstimM (Table 3). The differences of the RVP values measured by IOPstim minus measured by CLD were − 13.0 ± 5.6 mmHg.

**Table 2:** Retinal venous pressure values measured by contact lens dynamometry (CLD) and with IOPstim at an airway pressure of 20 mmHg. For each method, 3 readings were taken in rapid succession. Abbreviations: *CLD* contact lens dynamometry, *IOPstim* IOP stimulator, *Min* minimum, *Q1* 1st quartile, *Med* median, *Q3* 3rd quartile, *Max* maximum, *IQR* interquartile range

*N* = 36							
	Reading	Min	Q1	Med	Q3	Max	IQR
CLD	1	22.2	34.6	37.6	40.4	53.3	6.1
	2	21.3	35.2	37.3	41.0	48.4	5.8
	3	19.6	34.5	38.2	40.4	47.3	5.9
IOPstim	1	15.0	21.2	24.9	29.6	36.8	8.4
	2	15.8	21.2	24.7	28.3	34.5	7.1
	3	15.3	21.0	24.0	28.1	33.8	7.0

**Table 3:** Median range of the three measurements of the retinal venous pressure taken with the two methods at an airway pressure of 20 mmHg

*N* = 36						
Method	Min	Q1	Med	Q3	Max	IQR
CLD	1.0	2.2	2.7	4.4	8.8	2.2
IOPstim	0.8	2.2	2.9	3.4	6.2	1.3

When the CLD or the IOPstim were attached to the globe, a force was needed. The median IOP increase induced by that force (Table 4) was 10.0 mmHg with the CLDM and 2.2 mmHg with the IOPstimM (*p* < 0.001). The variability was higher with the IOPstimM.

**Table 4:** Increase in the intraocular pressure induced by positioning the instruments at the eye. For abbreviations, see Table 2

*N* = 36							
Instrument	Min	Q1	Median	Q3	Max	IQR	Wilcoxon test:* p* < 0.001
CLD	5.0	8.8	10.0	11.2	12.8	2.5	
IOPstim	− 6.0	− 0.3	2.2	5.4	16.2	5.6	

The median IOP decreased by 3.8 mmHg from baseline to the end of the experimental session. This difference was statistically significant (*p* < 0.001). For the CLDM, the RVP is defined as the sum of the IOP prior to the insertion of the CLD and the pressure increase induced by the instrument displayed on the LCD screen: RVP = IOP + ΔIOP.

**Fig. 5:**
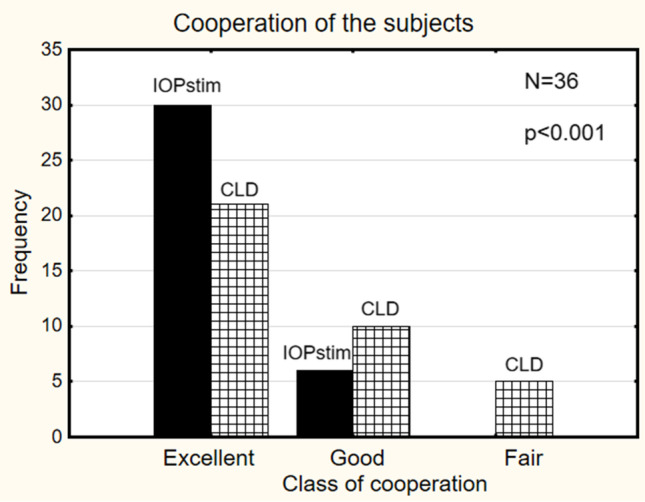
Frequency (ordinate) of classes corresponding to the level of cooperation of the subjects during the measurement

**Fig. 6:**
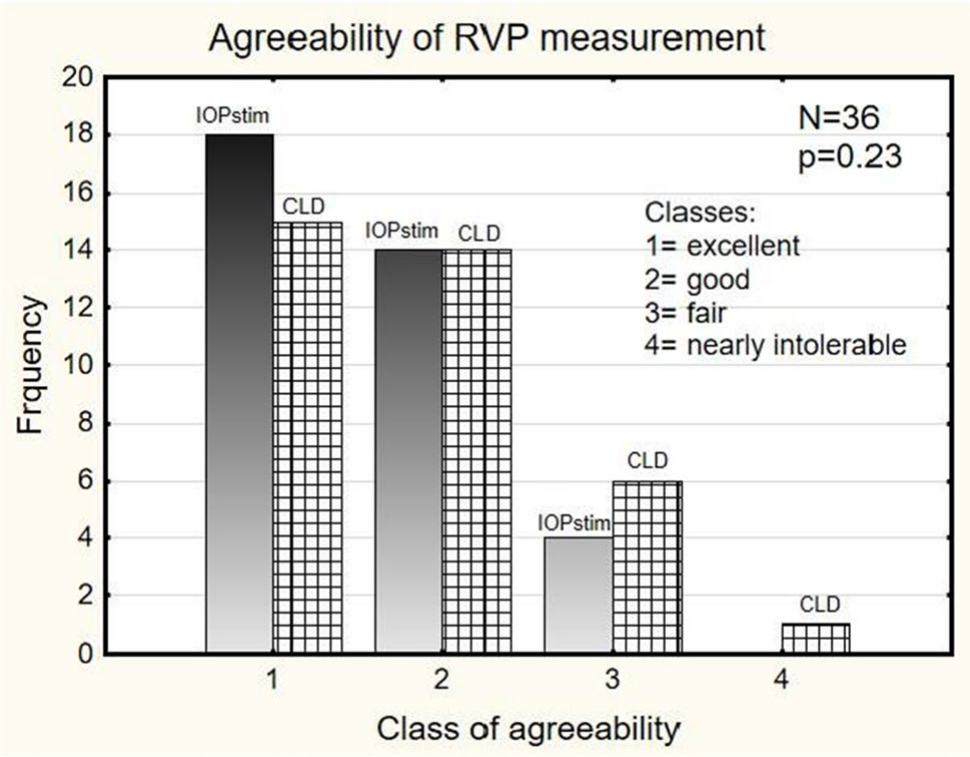
Agreeability of the subjects with the two different methods. Ordinate: frequency

The cooperation of the subjects was significantly better with the IOPstimM than with the CLDM (Fig. 5). The agreeability of the subjects with the two methods, as assessed by a five-stage classification system, did not differ significantly (*p* = 0.23). The measurement did not need to be interrupted because of pain for any of the subjects (Fig. 6). Twenty-four of the 36 subjects preferred the IOPstim for comfort, and the examiner reported that in 21 of the 36 subjects, the IOPstim examination was easier to execute.

## Discussion

### Reliability

The first question of the study was as follows: what is the reliability of the measurements? To answer this question, we calculated the median differences of the measurement values across the three consecutive time points of measurement for each of the two methods. Figure 4 shows that with the CLDM, there was a slight decrease by less than 2 mmHg from the first to the third measurement. For the IOPstimM, there was a minor increase in the median from the second to the third time point, which may be attributed to an increase in the range to higher values. Overall, the differences in the medians were minor in relation to the range shown by the box plots in Fig. 4 and in Table 3. This wide range may be attributed to the fact that all measurements of RVP were performed during the VM with an airway pressure of 20 mmHg. In the CLD measurements during the VM at an AirP of 20 mmHg in 42 subjects, the range of three readings had the same order of magnitude: 2.2 (1.8) mmHg) (median (interquartile range)). In 16 glaucoma patients, however, the CLD measurements (unpublished) without the VM showed a lower median of 1.5 (1.4) mmHg for the readings. Thus, it may be assumed that a considerable part of the variability of the RVP values in this study may be due to inter-individual effects of the AirP on the RVP caused by the peculiarities of the venous system [9].

Despite this wide range, the reliability showed good agreement, with ICC of 0.88 with the CLDM and 0.89 with the IOPstimM.

### Cooperation

The second question was as follows: would subjects cooperate with the measurement process? The answer for the IOPstim is shown in Fig. 5: in 30 of 36 subjects, the lids were fully slack, and in the remaining four subjects, the lid tension was low. For comparison, in 21 of the subjects for whom the CLD was used, the lids were fully slack, and in 10 subjects, contact was sensed, but the severity was tolerable. In five subjects, contact with the CLD was poor and tolerable for some minutes only. In comparison with the contact lens method used in daily clinical diagnostics, the IOPstim is well tolerated, and the cooperation is even better than that with the CLD.

### Agreeability

After the development of the IOPstim, the urgent question was: would subjects or patients tolerate measurements with the instrument? Therefore, we prepared a questionnaire: according to Fig. 6, eighteen subjects reported sensation after contact with the IOPstim but felt no irritation. An additional fourteen subjects distinctly sensed the contact and tolerated it well. Four subjects sensed the contact as poor but could tolerate it during the measurement. For comparison, the agreeability of the previously used CLDM was slightly less than that of the IOPstim. The difference was not statistically significant (Fig. 6).

### Why were measurements performed during the Valsalva manoeuvre?

The present study is the first to assess the applicability of the IOPstim in humans. For ethical reasons, the study was planned in healthy subjects. The vast majority of healthy subjects, however, show spontaneous pulsation of the CRV [10, 11], which indicates that the retinal venous pressure is equal to or slightly higher than the intraocular pressure [12, 13]. In these subjects, tonometry is sufficient to obtain an estimate of the RVP. Thus, a study in healthy subjects without this characteristic would probably have failed because of an insufficient number of eligible volunteers. When the RVP rises by 15 mmHg during the VM, no spontaneous pulsation of the CRV—a prerequisite of the measurement of RVP—can be observed. Blood pressure measurement at the upper arm is not possible if there is Korotkoff noise at a cuff pressure of zero. Despite the wide variability of the RVP values (interquartile range: 18 mmHg) in the presence of an AirP of 20 mmHg, we decided to conduct the measurements in the presence of a high AirP because the coefficient of variation was acceptable, with a mean of 8.1% [14].

### Peculiarity in RVP measurement

The *arterial* pressures measured by dynamometry [15] are generally considerably higher than the IOP. In these cases, the small IOP increase caused by the initial contact of the instrument with the globe does not affect the measurement. In the *RVP* measurement, however, different conditions exist. In cases of no pulsation of the CRV, pulsation may occur after the attachment of the CLD, even with the slightest possible force. The reason may be that the small IOP increase caused by this attachment leads the RVP to exceed the threshold. In these cases, the amount by which the value exceeds the threshold is unclear. This induced pressure increase is dependent on the lid tension, and the median was 10.0 mmHg with the CLDM and 2.2 with the IOPstimM (Table 4). The variability, however, described by the interquartile range, was considerably smaller with the CLDM. The negative values seen with the IOPstimM may also be due to the variability caused by tonometry. It can be expected that smaller RVP values would be recorded using the IOPstim than using the CLD.

### Changes in IOP

The IOP after dilation of the pupil was 1.8 mmHg lower than the initial value and remained 2.0 mmHg lower after the examinations. This last pressure decrease must be attributed to the so-called tonographic effect, which decreases the IOP. In RVP measurement, the threshold pressure is defined as the sum of the pre-existing IOP and the artificially induced increase in IOP. If the pre-existing IOP is low, a larger artificially induced increase in IOP is needed to reach the threshold pressure. This means that higher CLD readings may be caused by the tonographic effect. We used the IOP measured directly prior to the RVP measurement in the calculation of the RVP using the following equation: RVP = IOP + ΔIOP. For the IOPstimM, the tonographic effect does not play a role because the IOP is directly measured by tonometry.

### Higher RVP with the CLDM

An unexpected finding was that the median RVP values (Fig. 2 and Table 2) were 13 mmHg higher (*p* < 0.001) with the CLDM than with the IOPstimM. It may be discussed whether this difference may be caused by a calibration error in the *CLDM*. Morgan et al. [16], however, showed that calibration results were not significantly different from the CLD that we used [17]. Thus, a calibration error may be improbable. In addition, in this study, the median RVP values are 37.3–38.2 mmHg (Table 5) and hence in the same order of magnitude as in the healthy subjects in a different study in which the mean RVP was 35 mmHg at 20 mmHg of airway pressure [14]. This comparison may be a hint that the results of the CLDM in this study are not an exception.

**Table 5:** Intraocular pressure at three time points: baseline, after mydriasis and after the last IOP measurement. For abbreviations, see Table 2

*N* = 36						
Time point	Min	Q1	Med	Q3	Max	IQR
Baseline	11.8	12.2	16.4	18.1	24.4	2.9
After mydriasis	12.1	13.5	14.6	17.1	29.8	3.6
Last IOP	7.0	10.8	12.6	14.0	21.6	3.2

A calibration error in the *IOPstimM* is not probable because the artificially increased IOP during the measurement of the RVP was performed by the standardized iCare tonometer. The purpose of the IOPstim is only the increase of IOP and its maintenance during a short time span in which the increased IOP is measured.

A tonographic effect may be discussed in the IOPstimM because there is a maximal time span of 1 min between the observation of the pulsation and the measurement of the IOP. According to Ulrich and Ulrich, the pressure drop during this interval may approximately be 1.4 mmHg [18]. In this study, the IOPstim values were smaller by 13 mmHg than the values obtained by the CLDM. Thus, a possible tonographic effect may maximally contribute to this difference by 11%.

Another reason may be that we conducted measurements under Valsalva conditions, in which an increase in central (cerebral) venous pressure takes place [19]. These authors invasively measured the pressure in the jugular vein during the VM. In their investigations, this pressure was as high as the airway pressure. A congestion in the cerebral veins may therefore be assumed, as this congestion causes an additional pressure increase in the cerebral tissue. Because of the enclosure in the skull, the intracranial pressure may have also increased, which in turn compresses the CRV, increasing its resistance. The main vector with the CLDM is directed toward the apex of the orbit, where it contacts the congested tissue, additionally increasing its pressure. It may be hypothesized that this special condition causes the RVP to increase with the CLDM.

In contrast, with the IOPstimM, the main vector of the applied force is directed toward the medial wall of the orbit. Additionally, it may be assumed that the tissue pressure in the orbit is lower than in the skull during the VM because the frontal orbital wall is distensible. Thus, the resulting tissue pressure on the CRV outside the eye may be less with the IOPstimM than with the CLDM. Consequently, the CRV is associated with a smaller resistance and, therefore, a smaller pressure within the eye.

In the IOPstimM, the globe is deformed by the balloon. This condition may influence the physical properties of the cornea what in turn may alter the measurement values of the iCare tonometer. We cannot exclude this possibility but there are to the best of our knowledge no results in the literature which may back this hypothesis.

Whether the difference in the measurement values between the two methods is due to technical differences or due to the VM may be determined by the measurements without the VM. This investigation may be possible in glaucoma patients, as the SVP is not present in approximately 50% of these individuals [10, 20].

### Limitations

To test the new IOPstim in *healthy* volunteers, measurements had to be taken *during* the *VM*. As we pointed out, this condition may be the major reason for the significant change in the median by 13 mmHg.

The subjects included Caucasians only. The question of whether the IOPstim may also be applicable in subjects or patients with narrower palpebral fissures remains.

The judgments regarding cooperation and agreeability are subjective. Because the results are positive, we feel it justifiable to use the IOPstim in patients.

## Conclusion

The new IOPstim was well accepted by the subjects and by the examiner. The results show good reliability. The use of this instrument in clinical diagnostics seems justified. A further study in glaucoma patients is necessary in order to investigate whether the difference in the results of the two methods of measuring the RVP used here may also be present in glaucoma patients in which no VM is necessary because the pulsation of the central retinal vein is absent in about half of them.
